# Reproducibility assessment of biventricular strain derived from Long-Axis feature tracking in travelling Volunteers - A study in the Berlin research network for cardiovascular magnetic resonance (BER-CMR)

**DOI:** 10.1007/s10554-025-03540-5

**Published:** 2025-10-17

**Authors:** Clemens Ammann, Ralf Felix Trauzeddel, Maximilian Müller, Leonhard Grassow, Thomas Hadler, Darian Viezzer, Richard Hickstein, Leonora Zange, Edyta Blaszczyk, Elias Daud, Jeanette Schulz-Menger, Jan Gröschel

**Affiliations:** 1 Charité – Universitätsmedizin Berlin, corporate member of Freie Universität Berlin and Humboldt-Universität zu Berlin, Lindenberger Weg 80, 13125 Berlin, Germany; 2 Working Group on Cardiovascular Magnetic Resonance, Experimental and Clinical Research Center, a cooperation between Charité – Universitätsmedizin Berlin and the Max Delbrück Center for Molecular Medicine in the Helmholtz Association, Lindenberger Weg 80, 13125 Berlin, Germany; 3 Partner site Berlin, DZHK (German Centre for Cardiovascular Research), Berlin, Germany; 4 Department of Cardiology and Nephrology, HELIOS Hospital Berlin-Buch, Schwanebecker Chaussee 50, 13125 Berlin, Germany; 5 Department of Anaesthesiology and Intensive Care Medicine, Charité – Universitätsmedizin Berlin, corporate member of Freie Universität Berlin und Humboldt-Universität zu Berlin, Campus Benjamin Franklin, Hindenburgdamm 30, 12203 Berlin, Germany; 6 Helios IT Service GmbH, Schwanebecker Chaussee 50, 13125 Berlin, Germany; 7 The Cardiology Department, Galilee Medical Center, Azrieli Faculty of Medicine Bar-Ilan University, Nahariya - Safed, Israel; 8 Department of Cardiology, Angiology and Intensive Care Medicine, Deutsches Herzzentrum der Charité – Medical Heart Center of Charité and German Heart Institute Berlin, Charitéplatz 1, 10117 Berlin, Germany

**Keywords:** Cardiovascular magnetic resonance, Strain imaging, Feature tracking, Myocardial function, Travelling volunteers

## Abstract

**Purpose:**

To evaluate the reproducibility of biventricular global longitudinal strain (GLS) assessment using cardiovascular magnetic resonance in a multicenter study of travelling volunteers.

**Methods:**

Twenty travelling volunteers were prospectively scanned at four sites with same-vendor scanners at 3.0T (sites I, II, III) and 1.5T (site IV). Cine imaging in three long-axis views was performed using a segmented balanced steady-state free precession sequence with 30 cardiac phases except site II with 25 phases.

**Results:**

Imaging and post-processing were carried out successfully for 18 volunteers in a core lab setting. Pairwise comparisons revealed significant differences in left ventricular (LV) GLS between sites I and II (*p* < 0.001) and sites II and IV (*p* = 0.013), as well as in right ventricular (RV) GLS between sites I and IV (*p* = 0.027). RV GLS values were significantly higher at 3.0T (*p* = 0.024), whereas field strength had no significant impact on LV GLS (*p* = 0.153). Conversely, the use of 25 cardiac phases at site II was associated with significantly lower LV GLS values (*p* < 0.001), while RV GLS remained unaffected (*p* = 0.825).

**Conclusion:**

When applying feature tracking-based strain in a multicenter study, careful consideration should be given to the temporal resolution for LV longitudinal strain and to magnetic field strength for RV longitudinal strain.

**Graphical Abstract:**

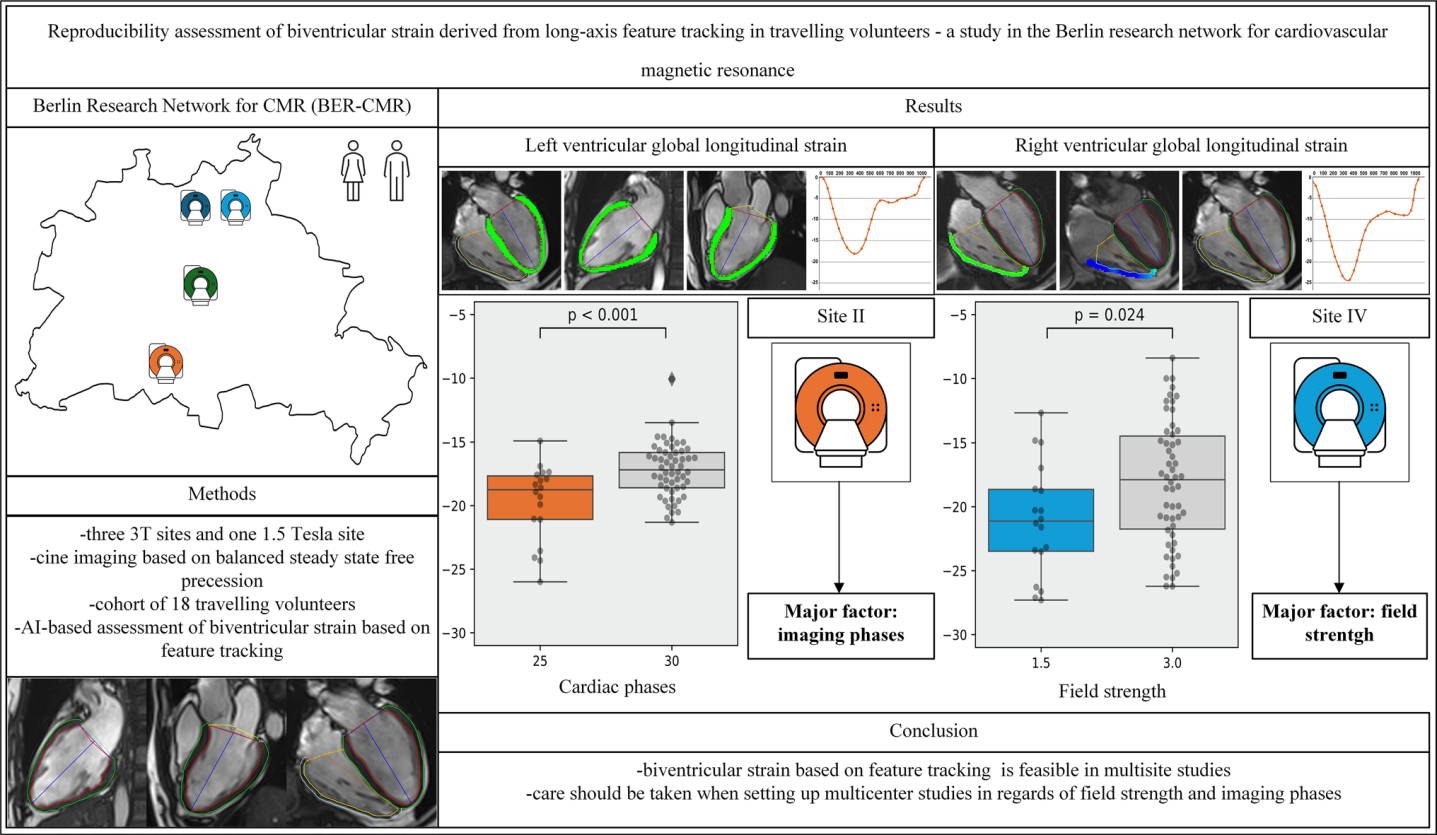

**Supplementary Information:**

The online version contains supplementary material available at 10.1007/s10554-025-03540-5.

## Introduction

Cardiovascular magnetic resonance (CMR) is a versatile imaging tool in the field of cardiology, providing detailed insights into cardiac function, morphology and tissue composition [1–3]. Therefore, its application in clinical trials has expanded, with large cohort studies, such as the UK Biobank or the German National Cohort (NAKO), incorporating CMR into the imaging protocol [4–6]. In addition, clinical endpoints are increasingly based on imaging markers derived from CMR [7]. With the growing focus on early disease detection and prevention, imaging markers that capture subclinical stages of pathologies are highly valuable and sought after [8, 9]. Strain imaging has been shown to capture reverse tissue remodeling at an early stage [10].

Global longitudinal strain (GLS) measures peak systolic shortening and is a strong predictor of mortality in acute heart failure [11] and adverse clinical outcomes in heart failure with preserved ejection fraction [12]. It has been successfully applied in the SUCCOUR-MRI (Strain sUrveillance of Chemotherapy for improving Cardiovascular Outcomes) trial to assign patients with anthracycline therapy to cardioprotection before ejection fraction deteriorates [13].

Strain assessment in CMR can be carried out using dedicated sequences, such as displacement encoding with stimulated echoes (DENSE) [14], or on routinely acquired balanced steady-state free precession (bSSFP) cine images [15]. Feature tracking (FT) is applied in image post-processing to track the myocardial borders for the assessment of left and right ventricular (LV and RV, respectively) strain measurements. Starting from a reference image with endo- and epicardial contours, anatomical features and intensity patterns (e.g. the blood-tissue border) are followed from frame to frame using optical flow and pattern recognition techniques [16, 17]. From the resulting displacement fields, segmental and global strain parameters, such as GLS, are derived. Main confounding factors for the analysis include the temporal resolution, the post-processing software, and reader experience [18–21]. Although studies have shown good agreement between different field strengths and scan-rescan comparisons [22, 23], no data exist on multisite comparisons with a travelling volunteer cohort.

The Berlin Research Network for CMR (BER-CMR) is a multisite project with the primary objectives to identify confounders that lead to differences in measurements and to establish a scanner platform for prospective clinical trials [24–26]. This study aims to analyze the reproducibility of biventricular GLS across the BER-CMR with a travelling volunteer cohort.

## Materials and methods

### Study protocol

The overall structure of the BER-CMR has been described in previous publications [24–26]. For this analysis, a cohort of 20 healthy volunteers was prospectively scanned at four sites of the BER-CMR with scanners from the same vendor (Siemens Healthineers, Forchheim, Germany). This included three sites equipped with a 3.0 T scanner (sites I with a Skyra^FIT^ and sites II and III with a Prisma^FIT^), while site IV used a 1.5 T scanner (Avanto^FIT^). Cine imaging was based on a bSSFP cine sequence with the following scan parameters at the 3.0 T sites: repetition time 38.4 ms, flip angle 52–59°, echo time 1.4 ms, field of view 301–322 × 360 mm, acquisition matrix 122–139 × 208, voxel size 1.7 × 1.7 × 6.0 mm. For the 1.5 T site they were: repetition time 33.0 ms, flip angle 59°, echo time 1.2 ms, field of view 292 × 360 mm, acquisition matrix 156 × 192, voxel size 1.9 × 1.9 × 6.0 mm. At all sites, 30 cardiac phases were acquired except for site II, for which 25 phases were acquired. Acquisitions were carried out in three long-axis views (LAX) including a four-chamber, a three-chamber and a two-chamber view [27]. At site I, a scan-rescan was performed with a 15-minute break between scans and a new positioning of the proband to assess tolerance intervals for equivalence testing [28]. Blood pressure and heart rate were measured electronically in supine position before each scan. The date and time of each scan was noted for each participant.

## Post-processing and strain analysis

Post-processing was carried out with dedicated software (cvi42 version 5.13.7, Circle Cardiovascular Imaging, Calgary, Canada) in a core lab setting at a single site by a single reader who was blinded to the examinations. Artificial intelligence (AI)-assisted cardiac segmentation was performed via batch processing as described recently [15]. GLS for the LV was based on FT in all three LAX views after endocardial and epicardial segmentation in end-diastole and end-systole. GLS for the RV was assessed by FT in the four-chamber view from endo- and epicardial segmentation at end-diastole. The implementation of the FT method used is based on a nearly incompressible deformable model, providing a physiologically plausible approximation of myocardial motion [29]. LV biplanar function values and volumes were derived from the four- and two-chamber views. All segmentations for function and strain analyses were visually assessed for quality control [15].

### Statistical analysis

Values are presented as median and interquartile range (IQR). Normal distribution was assessed with the Shapiro-Wilk test. Mean differences between sites were assessed using analysis of variance (ANOVA) for repeated measurements. In cases of significant differences, a post-hoc comparison using a paired two-sided t-test was caried out with a Bonferroni correction applied for multiple testing. To test for differences in field strength and temporal resolution, independent two-sided t-tests were performed. A p-value less than 0.05 was considered significant. To isolate the inter-site influence on mean deviations, tolerance intervals were established as ± 1.96 standard deviations from scan-rescan differences at site I. Equivalence was assumed if the 95% confidence of the mean bias between two sites remained within the respective tolerance interval [28]. Bland-Altman analysis was carried out to assess scan-rescan differences at site I. Statistical analysis was performed using SPSS (SPSS version 29, International Business Machines).

## Results

### General characteristics

From the initially recruited 20 volunteers, *N* = 18 underwent successful cine imaging at all sites. Two probands were excluded due to missing acquisitions at one site each. The cohort comprised seven women and eleven men with a median [IQR] age of 25.0 [22.0, 30.3] years, height of 1.81 [1.75, 1.85] m and weight of 71.5 [57.8, 79.3] kg. All volunteers were healthy at time of inclusion, but one subject was diagnosed with mild arterial hypertension shortly after inclusion. Median arterial systolic and diastolic blood pressure for the cohort were 119.0 [113.0, 127.0] mmHg and 71.0 [61.0, 80.0] mmHg, respectively. Median heart rate at time of scan was 67.5 [60.0, 72.3] beats/minute. The median difference of maximal and minimal blood pressure between scans was 15.0 [10.0, 21.0] [10, 21] mmHg systolic and 17.0 [11.0, 25.0] [11, 25] mmHg diastolic. Comparison of hemodynamic parameters revealed no significant differences. A median time interval of 14.0 [3.0, 47.0] days was noted between the first and last scans. The maximal time interval was 124 days, and the minimal time interval was three days (Supplementary Table 1).

### Quality assurance

**Fig. 1:**
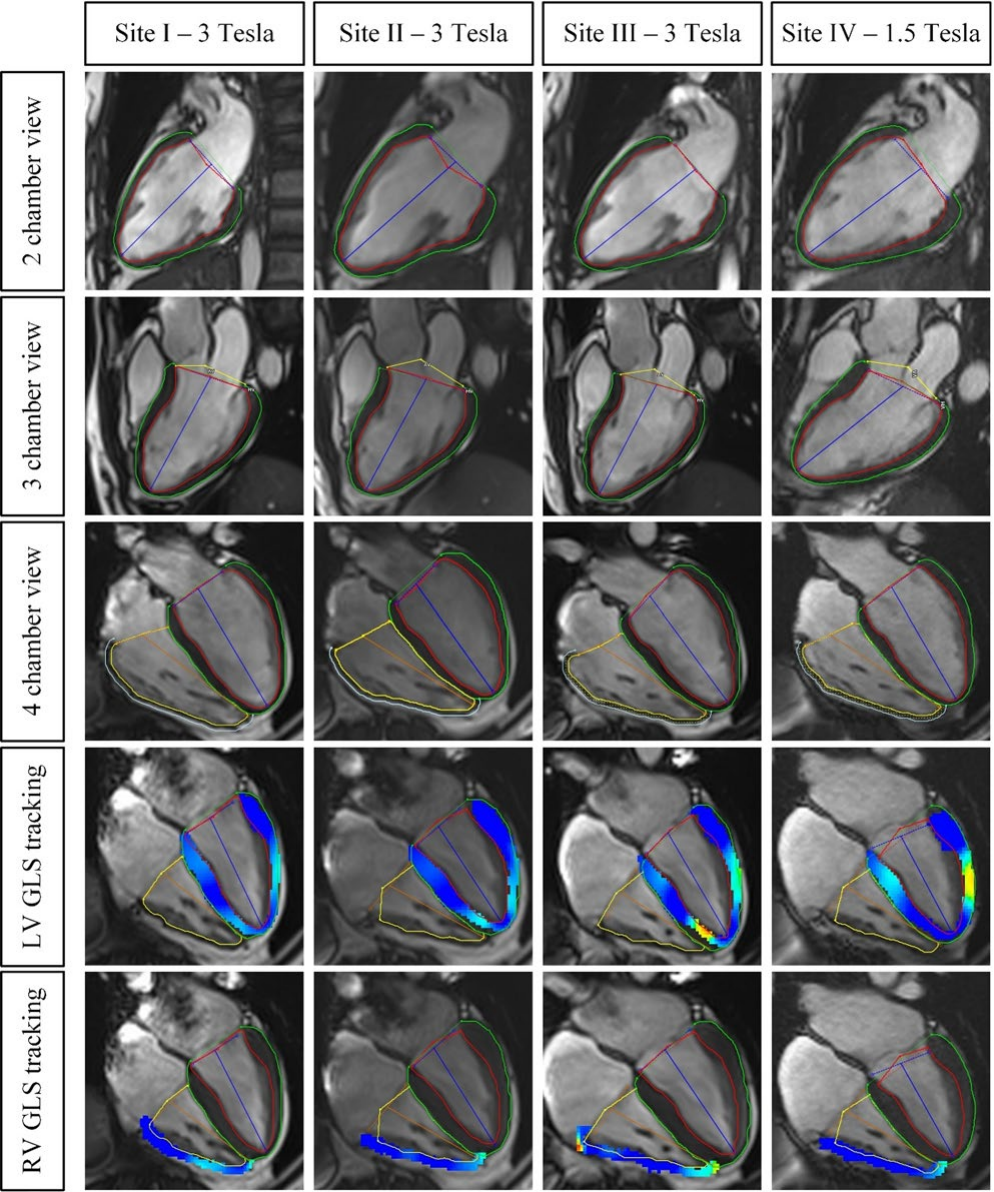
Examples of feature tracking-based left and right ventricular global longitudinal strain assessment for one volunteer across all sites. LV: left ventricle; GLS: global longitudinal strain; RV: right ventricle

Upon visual inspection, AI-based segmentation did not warrant manual corrections. Example images are shown in Fig. 1.

### Function, volume and mass analysis

Median values across all sites were as follows: LV end-diastolic volume 171.1 [153.7, 211.2] ml, LV stroke volume 97.0 [89.8, 120.1] ml, LV ejection fraction 57.3% [55.2%, 59.8%], LV mass 109.9 [94.5, 119.6] g. We observed a significant difference in LV ejection fraction across the sites (*p* = 0.002), with pairwise comparisons revealing a significant bias between sites II and III (*p* = 0.039), and sites II and IV (*p* = 0.002) (Table 1). However, when testing for mean differences applying published tolerance intervals for left ventricular ejection fraction [28], equivalence was assessed for all comparisons (Supplementary Fig. 1).

**Table 1:** Function and strain parameters across the sites

Parameter	Site I	Site II	Site III	Site IV	*p*-value
LV EDV (ml)	163.3 [149.0, 220.0]	173.7 [155.0, 211.9]	172.1 [153.6, 216.3]	178.8 [152.8, 210.3]	0.733
LV SV (ml)	94.7 [82.6, 126.0]	97.0 [89.1, 121.6]	100.8 [90.6, 116.2]	101.0 [90.9, 122.0]	0.112
LV EF (%)	56.3 [54.5, 59.9]	55.7 [53.1, 57.6]	57.9 [56.5, 59.8]	59.3 [57.4, 61.3]	**0.002 (II vs. III p = 0.039;** **II vs. IV p = 0.002)**
LVM (g)	112.9 [93.8, 133.6]	110.3 [93.9, 131.8]	112.3 [94.0, 129.2]	107.4 [91.7, 129.2]	0.579
LV GLS (%)	−16.4 [−18.4, −15.1]	−18.8 [−21.7, −17.5]	−17.7 [−19.6, −16.2]	−17.1 [−18.4, −15.8]	**< 0.001 (I vs. II p < 0.001;** **II vs. IV p = 0.013)**
RV GLS (%)	−16.9 [−21.8, −14.2]	−19.1 [−22.4, −15.8]	−18.1 [−22.3 −13.3]	−21.1 [−24.2, −18.2]	**0.032 (I vs. IV p = 0.027)**

All values are median and interquartile range. LV: left ventricle; EDV: end-diastolic volume; SV: stroke volume; EF: ejection fraction; LVM: left ventricular mass; GLS: global longitudinal strain; RV: right ventricle. Significant values are in bold

### Strain analysis

**Fig. 2:**
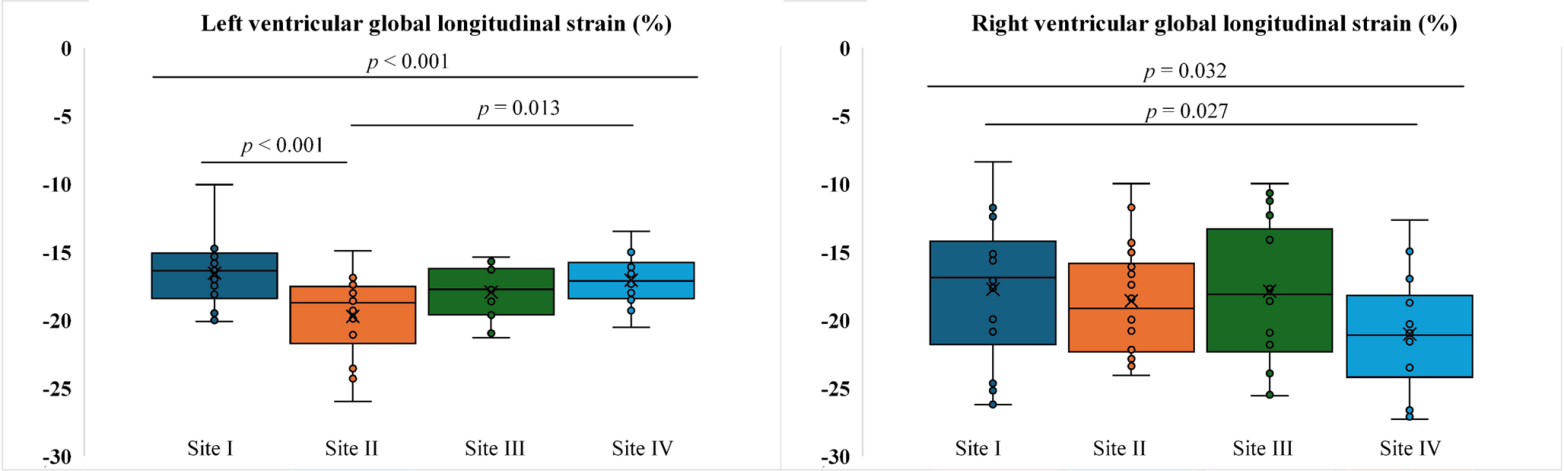
Boxplots for left and right ventricular global longitudinal strain across the BER-CMR. Boxplots represent median (solid line inside the box), interquartile range (IQR, box) and Q1–1.5×IQR or Q3 + 1.5×IQR (whiskers) for (A) left and (B) right ventricular global longitudinal strain at each site (site I: 3 T, site II: 3 T, site III: 3 T, site IV: 1.5T). p-values: one-way ANOVA (overall); t-test (pairwise; for significant comparisons). BER-CMR: Berlin Research Network for Cardiovascular Magnetic Resonance

**Fig. 3:**
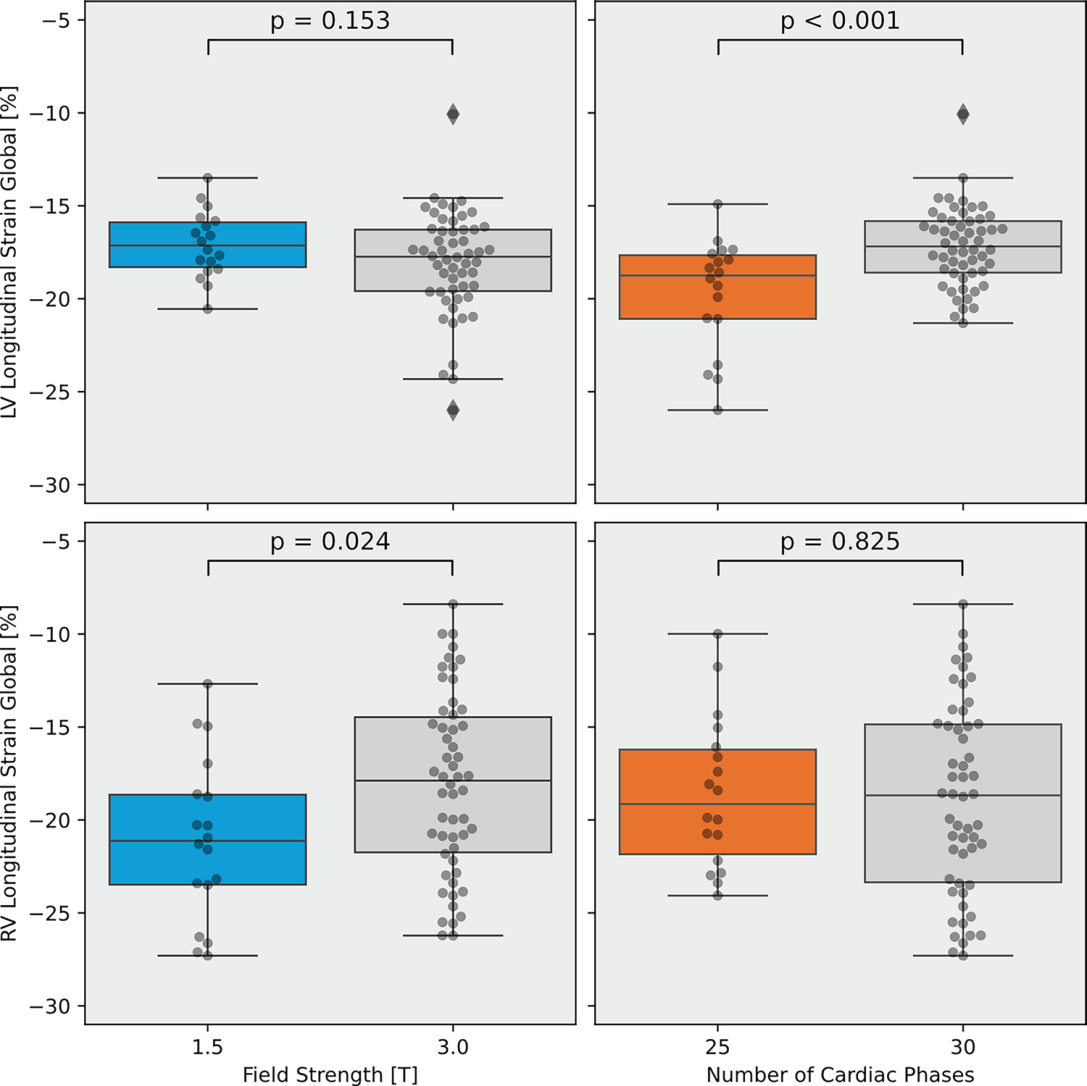
Boxplots for left and right ventricular global longitudinal strain by imaging phases and field strength. Boxplots represent median (solid line inside the box), interquartile range (IQR, box) and Q1–1.5×IQR or Q3 + 1.5×IQR (whiskers). p-values: independent t-test

The median value for LV GLS across all sites was − 17.6% [−19.4%, −16.2%], and for RV GLS − 18.6% [−21.7%, −14.9%]. Significant differences for LV GLS were found between sites I and II (site I −16.4% [−18.4%, −15.1%] vs. site II −18.8% [−21.7%, −17.5%]); *p* < 0.001) as well as sites II and IV (site II −18.8% [−21.7%, −17.5%] vs. site IV −17.1% [−18.4%, −15.8%]; *p* = 0.013). (Table 1; Fig. 2). For RV GLS, significant differences were detected between sites I and IV (site I −16.9% [−21.8%, −14.2%] vs. site IV −21.1% [−24.2%, −18.2%]; *p* = 0.027) (Table 1; Fig. 2). While there was no significant difference for LV GLS across field strength (*p* = 0.153), RV GLS values were significantly higher at the 3.0 T sites in comparison to the 1.5 T site (3.0 T −17.9% [−21.9%, −14.3%] vs. 1.5 T −21.1% [−24.2%, −18.2%]; *p* = 0.024) (Fig. 3). In contrast, LV GLS values were significantly lower with 25 cardiac phases than with 30 phases (25 phases − 18.8% [−21.7%, −17.5%] vs. 30 phases − 17.2% [−18.6%, −15.8%]; *p* < 0.001) but phase difference had no impact on RV GLS (*p* = 0.825) (Fig. 3). Scan-rescan differences at site I are reported in Supplementary Fig. 2 and were not significant for LV GLS (*p* = 0.136) and RV GLS (*p* = 0.768).

### Equivalence testing

**Fig. 4:**
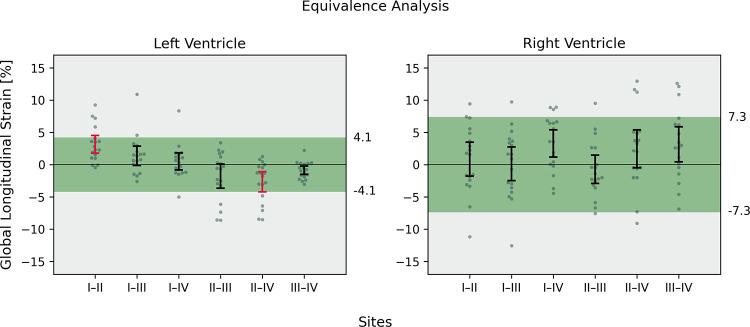
Equivalence testing for left and right ventricular global longitudinal strain across all sites. The underlying tolerance intervals (green areas) were derived from scan-rescan comparisons at site I (± 1.96 standard deviations of the difference). Error bars represent the 95% confidence interval for the mean bias from inter-site comparisons. Equivalence is assessed if the error bar lies completely within its corresponding tolerance interval (true for all comparisons except site I vs. II and site II vs. IV for left ventricular global longitudinal strain)

Tolerance intervals for biventricular strain assessment based on a scan-rescan at site I were established as: LV GLS ± 4.1%, RV GLS ± 7.3%. Equivalence was demonstrated for all parameters except LV GLS between sites I and II, and sites II and IV (Fig. 4).

## Discussion

In this sub-analysis of the BER-CMR travelling volunteer cohort, we demonstrate that the reproducibility of biventricular GLS assessment in multicenter studies is impacted by magnetic field strength for RV GLS and temporal resolution for LV GLS.

Strain assessment is a valuable parameter in cardiac imaging that can help to identify subclinical changes and patients at risk [30]. Potentially, it can be used to initiate early and preceptive treatment strategies. One such approach is the HERZCHECK study currently analyzing the value of GLS in patients at risk [7], with outcomes pending. Validation and confounder analysis is essential for any biomarker used in research or clinical settings. Recent studies analyzed the influence of field strength, temporal resolution, software vendor, scanner vendor and segmentation method on FT-derived strain [18–20, 22, 31–33]. While GLS was found to be overall reproducible, major confounders included the software used and the number of phases acquired with an overall agreement calling for a minimum of 30 phases [18]. In contrast to previous studies, this analysis comprised a travelling volunteer cohort, providing a robust foundation for inter-site agreement. We could confirm prior findings by showing inter-site differences due to differing temporal resolution, thus confirming the minimal requirement of 30 cardiac phases [18]. When testing for equivalence, two out of three inter-site comparisons for LV GLS at varying numbers of phases were outside of their respective tolerance range. For the single comparison (sites II and III) that remained inside, the exact same scanner model was used, suggesting that the specific scanner hard- and software may also contribute to the observed differences. While our work showed no significant effect of field strength on LV GLS, the literature on this topic is ambivalent, with some studies reporting an impact [34], and others finding no significant differences [20].

Our observation that fewer cardiac phases resulted in lower (more negative) LV GLS values is unexpected, as previous work described an increase in absolute strain at higher temporal resolution [18, 19]. Theoretically, a reduced number of cardiac phases may miss the true end-diastole and end-systole or impair feature tracking accuracy due to increased inter-frame pattern displacements. However, there are possible explanations for our finding. The reduced number of time points requires stronger interpolation over the cardiac cycle, effectively smoothing and regularizing the strain curve. This in turn may allow tracking algorithms to work more stably and result in apparently improved peak systolic strain estimates. Furthermore, variability in the selection of end-diastole and end-systole could have also contributed to differences in GLS values. In any case, increasing the temporal resolution beyond the limitation of the spatial resolution does not provide additional benefit for strain imaging, as movements less than one voxel cannot be captured [18].

While LV GLS is increasingly being applied in clinical and scientific settings, studies on RV GLS are still very limited. This study demonstrates that RV GLS values can be consistently measured across different sites with mean differences for all comparisons lying within their respective tolerance ranges. However, the wider tolerance interval demonstrates already greater variability for same-site reproducibility. Median RV GLS values in this study were slightly higher than in a recent meta-analysis, which showed a median RV GLS of −24.0% [34]. The same meta-analysis found that the software used for analysis has a major impact on RV GLS, with cvi42 yielding the highest values, which could explain the difference. Noteworthy, RV GLS differed between field strengths, which may be the main reason for the differences between sites I and IV. A prior study investigated the impact of field strength, spatial resolution and imaging sequence [35]. The authors concluded that RV GLS assessment across 1.5 T and 3.0 T with a bSSFP sequence shows good correlation with narrow limits of agreement [35]. Similar results were presented by Schuster et al. comparing 10 volunteers scanned on both 3.0 T and 1.5 T systems [22]. Our results contradict these findings and warrant further investigation. Possible explanations include the increased signal-to-noise ratio at 3.0 T or the slightly higher in-plane resolution of 1.7 × 1.7 mm at 3.0 T compared to 1.9 × 1.9 mm at 1.5 T in this study, which could particularly affect RV measurements due to the thinner wall compared to the LV. However, in previous works, spatial resolution was found to have no significant impact on strain [18].

Previous studies have shown that cardiac loading conditions, and the associated pre- and afterload, influence strain results [36]. High blood pressure may negatively impact LV strain [37]. In contrast, RV strain may be affected by preload, which can already change relevantly during breath holds for cine image acquisition [38]. Therefore, the integration of blood pressure and strain into a new index for “myocardial work” is proposed to better account for hemodynamic conditions in strain analysis [39]. In the current study, we recorded blood pressure measurements before image acquisition but not during the scan. We did not find any significant difference in median hemodynamic parameters between sites. In patient cohorts, blood pressure dysregulations may result in a stronger influence of loading conditions on strain than in healthy volunteers. Furthermore, irregular breath holds, or cardiac arrhythmias may additionally impair reproducibility in patient cohorts [40].

### Limitations

This study reports findings based on a cohort of 18 healthy volunteers, facilitating optimal image quality and breath-holding. Therefore, our results may not fully translate to patient cohorts. Despite the small sample size, the extensive logistical effort involved in scanning the same volunteers at four sites supports its adequacy for evaluating the robustness of multisite strain measurements. The observed differences in temporal resolution and field strength cannot be reliably isolated from other inter-site differences, since only one site each had a deviating field strength or number of cardiac phases. Blood pressure was only measured before, but not during, the scans. Given the time interval between examinations, changes in loading conditions may have influenced the results, despite no significant differences in blood pressure between sites.

## Conclusions

Global longitudinal strain assessments in a multicenter setup require harmonized imaging protocols with closely aligned temporal resolution. For right ventricular GLS, images should be acquired at the same magnetic field strength.

## Supplementary Information

Below is the link to the electronic supplementary material.

Supplementary Material 1

## Data Availability

The datasets analyzed in the current study are not publicly available due to German laws but are available from the corresponding author on reasonable request.

## Abbreviations

AI: Artificial intelligence
bSSFP: Balanced steady-state free precision
GLS: Global longitudinal strain
LV: Left ventricle
CMR: Cardiovascular magnetic resonance
FT: Feature tracking
RV: Right ventricle
BER-CMR: Berlin Research Network for CMR
LAX: Long axis
AI: Artificial intelligence
IQR: Interquartile range

## Declarations

### Consents to participate

Informed consent was obtained from all individual participants included in the study.
