# Supplementary material for: Reproducibility assessment of biventricular strain derived from Long-Axis feature tracking in travelling Volunteers - A study in the Berlin research network for cardiovascular magnetic resonance (BER-CMR)
Source: Int J Cardiovasc Imaging. 2025 Oct 17;41(12):2353–61. doi: 10.1007/s10554-025-03540-5 (PMC12678522; doi:10.1007/s10554-025-03540-5)
Supplement: Supplementary file 1 — Supplementary Material 1 [file 10554_2025_3540_MOESM1_ESM.pdf]

Supplementary Material

Supplementary Table 1: Time intervals between scans.

Proband	Δ days 1st and 2nd scan	Δ days 2nd and 3rd scan	Δ days 3rd and 4th scan
1	2	1	1
2	2	1	1
3	1	1	34
4	1	1	34
5	1	1	1
6	1	1	1
7	6	1	14
8	1	112	11
9	1	1	1
10	1	1	49
11	1	1	1
12	5	1	2
13	1	2	1
14	3	5	16
15	1	2	0
16	6	8	89
17	47	22	7
18	5	54	1

Δ days: time difference in days

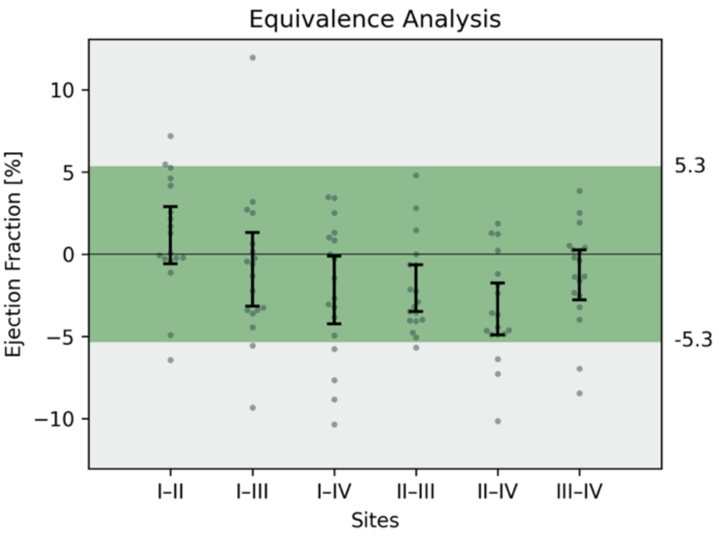

Supplementary Figure 1: Equivalence testing for left ventricular ejection fraction.
The underlying tolerance interval (green area) was published by Zange et al. [1]. Error bars represent the 95% confidence interval for the mean bias from inter-site comparisons. Equivalence is assessed if the error bar lies completely within its corresponding tolerance interval (true for all comparisons).

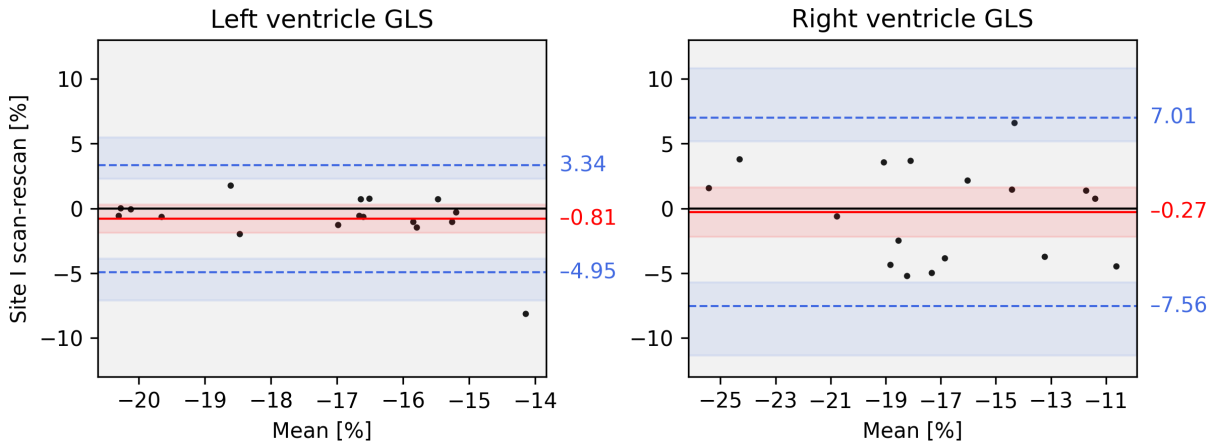

Supplementary Figure 2: Bland-Altman analysis for scan-rescan differences at site I.

GLS: global longitudinal strain

References

1. Zange L, Muehlberg F, Blaszczyk E, et al (2019) Quantification in cardiovascular magnetic resonance: agreement of software from three different vendors on assessment of left ventricular function, 2D flow and parametric mapping. J Cardiovasc Magn Reson Off J Soc Cardiovasc Magn Reson 21:12. https://doi.org/10.1186/s12968-019-0522-y
